# The effect of dry cow therapy using systemic tylosin in combination with common intramammary medications on mastitis rate, cull rate, somatic cell count, and milk production in dairy cows affected with subclinical mastitis

**DOI:** 10.14202/vetworld.2018.1266-1271

**Published:** 2018-09-15

**Authors:** Zuhair Bani Ismail, Mohammad Musab Muhaffel, Ehab Abu-Basha

**Affiliations:** Department of Veterinary Clinical Sciences, Faculty of Veterinary Medicine, Jordan University of Science and Technology, Irbid 22110, Jordan

**Keywords:** dairy cows, dry cow therapy, subclinical mastitis, tylosin

## Abstract

**Aims:**

This study was performed to evaluate the effect of systemic tylosin on mastitis rates, cull rates because of mastitis, and quality and quantity of milk production in dairy cows affected with subclinical mastitis.

**Materials and Methods:**

A total of 130 California mastitis test (CMT)-positive cows were randomly selected and divided into four different treatment groups. All treatments were performed on the day of drying off. Cows in Group 1 (n=34) received 12 g of tylosin intramuscularly (IM) and intramammary (IMM) 400 mg novobiocin sodium and 200,000IU penicillin G procaine. Group 2 (n=33) received 12 g tylosin IM and IMM 280 mg benethamine penicillin, 100 mg penethamate hydriodide, and 100 mg framycetin sulfate. Group 3 (n=33) received IMM alone with 400 mg novobiocin sodium and 200,000 IU penicillin G procaine. Group 4 (n=30) received IMM alone with 280 mg benethamine penicillin, 100 mg penethamate hydriodide, and 100 mg framycetin sulfate. The incidence and severity of clinical mastitis (CM), incidence of chronic mastitis, and cow cull rate because of mastitis were recorded during the first 100 days in milk (DIM). In addition, somatic cell count (SCC) and milk production parameters including the average days to peak milk yield, the average milk yield at peak, the average milk yield during the first 100 DIM, and the average 305-corrected milk yield were reported.

**Results:**

The rate of CM was significantly (p≤0.05) less in Group 2 when compared between the current and previous lactations (30% vs. 64%). In Group 1 and 4, the rate of CM was decreased but not significant between the two lactations (59% vs. 79% and 63% vs. 77%, respectively) while in Group 3, the rate of CM was slightly increased (82% vs. 91%). When compared between the four groups in the current lactation, CM rate was significantly (p≤0.05) less in Group 2 compared to the other groups. A significant (p≤0.05) percentage of CM cases in Group 2 was classified as mild. In Groups 1 and 3, a significant (p≤0.05) percentage of CM cases was classified as moderate while severe clinical signs were recorded more significantly (p≤0.05) in Groups 3 and 4. The rate of chronic mastitis was significantly less in Group 1 and Group 2 in the current lactation compared to that in the previous lactation (6% vs. 12% and 0% vs. 6%, respectively). In Groups 3 and 4, the rate of chronic mastitis was not changed significantly when compared between the current and previous lactations. No cows were culled because of mastitis in Groups 1 and 3 while one cow was culled in each of Groups 2 and 4 during the first 100 DIM in the current lactation. The average milk yield during the first 100 DIM and the 305-corrected milk yield were significantly (p≤0.05) increased in Group 2 when compared between the previous and current lactations. Furthermore, cows in Group 2 produced significantly (p≤0.05) more milk during the first 100 DIM and significantly (p≤0.05) more 305-corrected milk yield compared to the other groups. In Group 2, the average SCC dropped significantly (p≤0.05) from 1,600,000 cells/ml at the start of the study to <200,000 cells/ml at 100 DIM.

**Conclusions:**

In dairy herds with subclinical mastitis, dry cow therapy of CMT-positive cows using a combination of tylosin (12 g, IM) and IMM administration of benethamine penicillin, penethamate hydriodide, and framycetin sulfate (Ubrostar; Boehringer Ingelheim, Germany) may result in a significant reduction of the rate and severity of acute and chronic mastitis and cull rates due to mastitis within the first 100 DIM. Furthermore, treated cows may produce significantly more milk with less SCC during the first 100 DIM and therefore produce significantly more 305-corrected milk in the lactation following treatment.

## Introduction

Dry cow therapy using a combination of intramammary (IMM) and systemic administration of antibiotics is considered effective against invasive udder pathogens such as *Staphylococcus aureus* [1-7]. Tylosin, a member of the macrolides family of antimicrobials, has been approved for use in the treatment of mastitis in many parts of the world [3,8]. It is characterized by high lipid solubility and bioavailability, long half-life, and low protein binding [3,8]. Once administered systemically, tylosin quickly diffuses to the udder tissues resulting in very high milk-to-plasma concentration ratio [8,9].

Recently, there have been great concerns by consumers about antibiotic residues in milk and the growing threats caused by the emergence of antibiotic resistance among common human and animal pathogens [1,4]. In compliance with the World Health Organization recommendations, livestock industry is now accepting that antibiotics must only be used to treat infected animals. These efforts have led to the development of selective dry cow therapy (SDCT) [10]. In this context, SDCT focuses on the elimination of existing infections [7,10].

In this study, the effect of dry cow therapy using systemic administration of tylosin in combination with two different commonly used commercially available IMM dry cow therapy medications on the incidence of acute and chronic mastitis, cow cull rate because of mastitis, somatic cell count, and milk production in dairy cows affected with subclinical mastitis was evaluated. The theory here is that, in dairy farms with high rates of subclinical mastitis, the addition of tylosin to their dry cow therapy protocols using common dry cow therapy medications improves udder health and reduces the incidence of both acute and chronic mastitis in the following lactation, leading to the production of more milk of low somatic cell count and less use of antibiotics on the farm.

## Materials and Methods

### Ethical approval

All procedures used in this study were reviewed and approved by the Jordan University of Science and Technology Animal Care and Use Committee (JUST-ACUC).

### Animals

A total of 130 dairy cows were used in this study. The cows belonged to a single dairy farm located in North Eastern Jordan. The average number of cows on this farm was 550. Cows were Holstein-Friesian. The farm was selected for this study based on the farmers request to investigate a persistently high bulk tank somatic cell count and chronic mastitis problems. All cows were housed in free stall housing in dirt lots with shaded areas provided. The cows were offered a total mixed ration 3 times per day. Fresh water was offered *ad libitum*. Cows were milked in a double herringbone, high line milking parlor 3 times per day. Inconsistent teat preparation procedures and inadequate milking hygiene were practiced in the herd. Automatic milking machine takeoff and automatic machine flushing system were used.

### Subclinical mastitis determination and sample collection

California mastitis test (CMT) was performed on all cows in the herd using routine procedures. Quarter milk samples were collected from CMT-positive cows before treatment and repeated at 100 days in milk (DIM). Samples were kept on ice during transportation to the laboratory. Somatic cell count (SCC) was performed manually within 2 h after sample collection [11]. Briefly, a 0.01 ml of thoroughly mixed milk from each sample was spread on a 1 cm^2^ area on a glass slide. The slides were left to air dry on a flat surface and were stained by Newman-Lampert stain and examined microscopically. 20-40 fields were counted for each sample to ensure reproducibility and accuracy. A threshold of 250,000 cells/ml was considered a cutoff point for classification of subclinical mastitis in individual samples.

### Treatment allocation

Only CMT-positive cows were randomly selected and enrolled in the study (n=130). Cows were randomly divided into four different treatment groups and received one of the following treatment protocols on the day of drying off (Table-1): Group 1 (TAD; n=34) received 12 g of tylosin (Macrolan-200, Interchemie, The Netherlands) intramuscularly (IM) and IMM 400 mg novobiocin sodium and 200,000 IU penicillin G procaine (Albadry plus; Zoetis, New Jersey, USA). Group 2 (TUS; n=33) received 12 g tylosin IM and IMM 280 mg benethamine penicillin, 100 mg penethamate hydriodide, and 100 mg framycetin sulfate (Ubrostar; Boehringer Ingelheim, Germany). Group 3 (AD; n=33) received IMM alone with 400 mg novobiocin sodium and 200,000IU penicillin G procaine. Group 4 (US; n=30) received IMM alone with 280 mg benethamine penicillin, 100 mg penethamate hydriodide, and 100 mg framycetin sulfate.

**Table-1:** Number of cows, parity, group distribution, and treatment protocol of cows included in the study.

Groups	n	Parity	Treatment protocol
1	34	3.9±1.1	Tylosin 12 g IM IMM medication^≠^
2	33	3.8±1.2	Tylosin 12 g IM IMM medication^¥^
3	33	3.8±1.0	IMM^≠^alone
4	30	3.3±1.2	IMM^¥^ alone

IM=Intramuscular, IMM=Intramammary,

≠ 400 mg novobiocin sodium and 200,000IU penicillin G procaine (Albadry plus; Zoetis, New Jersey, USA).

¥ 280 mg benethamine penicillin, 100 mg penethamate hydriodide, 100 mg framycetin sulfate (Ubrostar; Boehringer Ingelheim, Germany)

### Pre and post drying-off procedures

The drying-off procedure was applied consistently to the cows involved in the study. Briefly, cows within 66-60 days to calving date were moved to the parlor to perform the last milking and prepare the teats for IMM infusion. The teat ends were cleaned and sanitized with germicidal pre-dip sanitizer (Keno Pure; CID LINES, Belgian) and then the teas were wiped off with a separate napkin. The workers who were performing the procedures wore protecting disposable latex gloves. Before infusion, the teat orifice was scrubbed carefully with a disposable tissue soaked with alcohol (70%) (separate tissue for each quarter). The test on the far side of the udder was cleaned first followed by the one close to the operator. For IMM infusion, only the tip of the cannula of the IMM tube was inserted into the teat orifice where all the contents were expressed. Post-dipping of the teats using a germicidal sanitizer (Keno Cidin; CID LINES, Belgium) was used after treatment. Tylosin (12 g) was injected IM in the neck region in cows in treatment Groups 1 and 2. Tylosin dose was split into two different sites. Cows were then identified with a colored ring on the leg and her electronic record updated using Alpro herd management software (DeLaval, New Zealand).

### Mastitis monitoring

Acute mastitis was defined as inflammation of one or more quarters associated with physical changes in milk secretion (consistency, color, and presence of clots), changes in udder size (swelling), udder pain or warmth, and systemic clinical signs (anorexia, fever, and toxemia). Cows were monitored for signs of clinical mastitis (CM) during the dry period and the first 100 DIM. Farm personnel and milking parlor operators were trained on the detection of CM and milk sampling techniques. In cases of CM, the number of affected cows was recorded, milk samples were collected, and treatment using routine CM protocols was initiated.

The severity of clinical signs associated with mastitis was scored as mild (only changes in milk consistency, presence of clot, and color), moderate (changes in milk secretion, and slight udder swelling, pain, and warmth), and severe (changes in milk secretion, slight udder swelling, pain and warmth, and systemic clinical signs).

Chronic mastitis was defined as cows with one or more quarters with frequent flare-ups of CM. The number of cows with repeated bouts of CM during the first 100 DIM was recorded.

### Cow cull rate

The number of cows culled, died, or removed from the production herd due to mastitis during the first 100 DIM was recorded.

### Milk production parameters

To assess the effect of various treatments on milk production, the following parameters were recorded: The average DIM to peak milk yield, average milk produced at peak, average milk produced during the first 100 DIM, and average 305-corrected milk yield.

### Statistical analysis

Descriptive analysis was carried out to explore the data using Excel software program (Microsoft Corp., Redmond, WA). Analysis of variance was used to compare the means between treatment groups. Treatment efficacy was performed using the Chi-square test to investigate differences in proportions between groups and between the previous and current lactations. Bonferroni test was used to allow for multiple comparisons where appropriate. Variables with p≤0.05 were considered statistically significant. The statistical analysis was performed using SPSS software version 23 (SPSS, USA).

## Results

The average parity of selected cows for this study was 3.7 (Table-1). The incidence of acute and chronic mastitis and cow cull rate because of mastitis during the first 100 DIM in the current and previous lactations are presented in Table-2. The rate of CM was significantly (p≤0.05) less in Group 2 when compared between the current and previous lactations (30% vs. 64%). In Group 1 and 4, the rate of CM was decreased but not significant between the two lactations (59% vs. 79% and 63% vs. 77%, respectively). In Group 3, the rate of CM was slightly increased in Group 3 (82% vs. 91%) in the current lactation compared to the previous one. When compared between the four groups in the current lactation, CM rate was significantly less in Group 2 (30%) compared to the other groups (Table-2). The rates of CM in Groups 1, 3, and 4 were 59%, 91%, and 63%, respectively.

**Table-2:** Incidence of acute clinical and chronic mastitis and cow cull rate due to mastitis during the first 100 DIM (%) in dairy cows with subclinical before and after receiving different IMM dry cow therapy with or without intramuscular injection of tylosin at drying off.

Groups	Previous lactation		Current lactation		Culled cows^¥^
	
	Acute mastitis	Chronic mastitis	Acute mastitis	Chronic mastitis	
1	27 (79)	4 (12)^a^	20 (59)	2 (6)^á^*	0
2	21 (64)^a^	2 (6)*	10 (30)^á^*	0	1 (3)
3	27 (82)	4 (12)	30 (91)	4 (12)	0
4	23 (77)	2 (7)*	19 (63)	2 (7)*	1 (3.3)

a =á indicate significant value at P≤0.05 between previous and current seasons,

* indicate significant value at P≤0.05 between different groups in the same season,

¥ cows culled, died, or removed from the production herd because of mastitis during the current lactation. DIM=Days in milk

In the first 100 DIM in the previous lactation, the number of chronic mastitis cases in the four groups was generally higher than the current lactation (12 cases vs. 8) (Table-2). The rate of chronic mastitis was significantly (p≤0.05) less in Group 1 and Group 2 in the current lactation compared to that in the previous lactation (6% vs. 12% and 0% vs. 6%, respectively). In Groups 3 and 4, the rates of chronic mastitis were not changed significantly when compared between the current and previous lactations.

**Figure-1:**
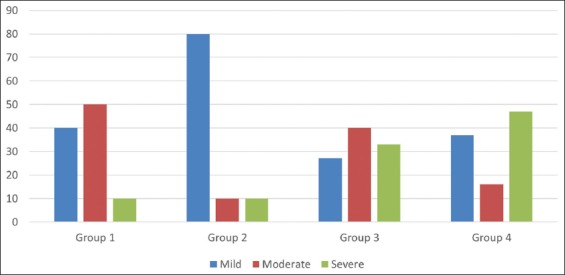
Severity of clinical mastitis in the first 100 days in milk (%) in dairy cows with subclinical mastitis before and after receiving different IMM dry cow therapy with or without intramuscular injection of tylosin at drying off.

The severity of CM recorded in different groups during the first 100 DIM is present in Figure-1. In a significant (p≤0.05) percentage of cases in Group 2, signs of CM were classified as mild. In Groups 1 and 3, a significant (p≤0.05) percentage of CM cases was classified as moderate while severe clinical signs were recorded more significantly (p≤0.05) in Groups 3 and 4. In Group 1, clinical signs of CM were classified as mild in 40% of cases, in 50% of cases as moderate, and in 10% of cases as severe. In Group 2, 80% of CM cases, clinical signs were classified as mild while 10% were classified as moderate and 10% were classified as severe. In Group 3, most of the cases were classified as moderate (40%) and severe (33%), while 27% of cases were classified as mild. In Group 4, most cases were classified as mild and severe CM (375 and 47%, respectively). Only 16% of CM cases were classified as moderate in this group.

The rate of cows culled, died, or removed from the production herd because of mastitis in the first 100 DIM in current lactation is present in Table-2. No cows were culled, died, or removed in Groups 1 and 3 while in Groups 2 and 4, two cows were culled, died, or removed because of mastitis (one each).

The mean±standard deviation of days to peak milk yield, milk yield at peak, milk yield during the first 100 DIM, and 305-corrected milk yield in the previous and current lactations are presented in Table-3. The average milk yield during the first 100 DIM and the 305-corrected milk yield were significantly (p≤0.05) increased in Group 2 when compared between the previous and current lactations. Furthermore, in the current lactation, cows in Group 1 produced significantly (p≤0.05) more milk at 100 DIM and 305-corrected milk compared to other groups. No significant differences were recorded in days to peak yields, average milk yield at peak, milk yield during the first 100 DIM, and 305-corrected milk yield between groups in the previous lactation.

**Table-3:** Days to peak milk yield, milk yield at peak, 100 DIM yields, and 305-corrected milk yield in the previous and current lactation in dairy cows with subclinical mastitis before and after receiving different IMM dry cow therapy with or without intramuscular injection of tylosin at drying off (means±SD; kg).

Groups	Previous lactation				Current lactation			
	
	Days to peak	Milk yield at peak	100 DIM yield	305-corrected milk yield	Days to peak	Milk yield at peak	100 DIM yield	305-corrected milk yield
1	48±22	45±7	2616±500	7979±1527	47±23	50±8	2600±650	8062±2223
2	44±15	45±8	2600±655^a^	8131±2170^a^	45±15	46±6	3100±49^á*^	9746±1537^á*^
3	52±16	43±6	2800±490	8664±1521	51±18	45±6	2900±490	8992±1502
4	46±14	43±7	2600±450	140±86400	46±15	45±6	2900±650	9040±1840

a=á indicate significant value at P≤0.05 between previous and current seasons,

* indicate significant value at P≤0.05 between different groups in the same season. DIM=Days in milk, SD=Standard deviation

The average SCC before the start of the study was 1,600,000±650 cells/ml. The average SCC dropped significantly (<200,000 cells/ml; p≤0.05) at 100 DIM in cows treated with systemic tylosin and IMM infusion of benethamine penicillin, penethamate hydriodide, and framycetin sulfate compared to other groups.

## Discussion

Mastitis remains one of the most common and economically devastating diseases affecting the dairy cow industry in Jordan [11-14] as well as elsewhere in the world [15-19]. In the herd under study here, the prevalence of subclinical mastitis was 39.3%. The prevalence of subclinical mastitis in Jordan has been reported to reach up to 94% in some studies [11-14]. This great variation in the prevalence of subclinical mastitis has been suggested to be due to differences in the methods of diagnosis (CMT vs. SCC), geographical differences, and differences in milking and environmental management between herds [11,12].

Tylosin, a member of the macrolides family of antimicrobials, has been used as a systemic treatment of CM caused by Gram-positive bacteria [3,6,8,9]. It has been determined that systemic administration of tylosin result in a rapid diffusion to the mammary gland resulting in high milk-to-plasma concentration ratio of 5:1 [3,8,9]. In this study, the systemic administration of tylosin along with the IMM administration of benethamine penicillin, penethamate hydriodide, and framycetin sulfate (Ubrostar; Boehringer Ingelheim, Germany) at drying off in dairy cows with subclinical mastitis resulted a significantly less episodes of acute mastitis during the first 100 DIM when compared between the current and previous lactations. Furthermore, the number and percentage of chronic mastitis cases were also significantly reduced. This indicates that tylosin was effective not only in eliminating existing IMM infections at drying off but also prevented new IMM infections. Similar results were reported previously when tylosin was administered systemically along with IMM infusion of cefapirin [9].

Moreover, in this study, the clinical signs of acute mastitis in a significant percentage in tylosin-treated cows (Groups 1 and 2) were classified as mild to moderate in severity in comparison to other groups. These results may indicate that most of the pathogens causing the mastitis in this study were sensitive to tylosin. These results are congruent with previously published data regarding the use of tylosin for the treatment of mastitis which has indicated that resistance in *S. aureus* causing mastitis in cows has not emerged and that resistance in *Streptococcus uberis* has remained stable [6]. Indeed, tylosin has been shown effective against the most common causative agents of mastitis, namely *S. aureus* and *S. uberis* in dairy cows [6]. The minimum inhibitory concentration of tylosin against *S. aureus* and *S. uberis* was reported to range from 0.25 to 2 μg/ml and 0.125 to >256 μg/ml, respectively.

Milk production parameters during the first 100 days of milk and 305-corrected milk production were significantly improved in cows after treatment with systemic tylosin. These results are in agreement with previous findings where acute and chronic mastitis were reported to result in a significant loss of milk production [20-22]. The improved udder health resulted in significantly higher milk production in the first 100 days of lactation and over the entire lactation in the cows treated with systemic tylosin combined with IMM injection of benethamine penicillin, penethamate hydriodide, and framycetin sulfate compared to other groups. The improved udder health in this group of cows was also shown by a significant reduction in the milk somatic cell count and fewer cows that were culled, died, or removed from the production herd because of mastitis.

## Conclusion

In dairy herds with subclinical mastitis, dry cow therapy of CMT-positive cows using a combination of tylosin (12 g, IM) and IMM administration of benethamine penicillin, penethamate hydriodide, and framycetin sulfate (Ubrostar; Boehringer Ingelheim, Germany) may result in a significant reduction of the rate and severity of acute and chronic mastitis and cull rates due to mastitis within the first 100 DIM. Furthermore, treated cows may produce significantly more milk with lower SCC during the first 100 DIM and therefore significantly more 305-corrected milk yield in the lactation following treatment.

## Authors’ Contributions

ZBI designed the experiment and wrote the manuscript. MMM conducted the experimental work. EAB edited the manuscript and performed data analysis. All authors read and approved the final manuscript.

## References

[1] Berry E.A, Hillerton J.E (2002). The effect of selective dry cow treatment on new intramammary infections. J. Dairy Sci.

[2] Bradley A.J, Green M.J (2004). The importance of the nonlactating period in the epidemiology of intramammary infection and strategies for prevention. Vet. Clin. Food Anim.

[3] Bonnier M, Dore C, Amedeo J, Guerin-Faublee V (2006). *In vitro* activity of tylosin and tilmicosin against cocci isolated from bovine mastitis. Rev. Med. Vet.

[4] Robert A, Seegers H, Bareille N (2006). Incidence of intramammary infections during the dry period without or with antibiotic treatment in dairy cows-a quantitative analysis of published data. Vet. Res.

[5] Pyörälä S (2008). Mastitis in post-partum dairy cows. Reprod. Dom. Anim.

[6] Simjee S, Amedeo J, Barletta A.M, Hogeveen H, Lam T.J.G (2011). Use of Tylan 200®for the treatment of mastitis caused by *Staphylococcus aureus* and/or *Streptococcus uberis*. Udder Health and Communication.

[7] Rajala-Schultz P.J, Torres A.H, DeGraves F.J (2011). Milk yield and somatic cell count during the following lactation after selective treatment of cows at dry-off. J. Dairy Res.

[8] McDougall S, Agnew K.E, Cursons R, Hou X.X, Compton C.R.W (2007). Parenteral treatment of clinical mastitis with tylosin base or penethamate hydriodide in dairy cattle. J. Dairy Sci.

[9] Contreras B.G.A, Guterbock W.M, Muñoz R.J.M, Sears P.M (2013). Comparison of systemic and intramammary dry cow treatments. Rev. MVZ Córdoba.

[10] Halasa T, Østerås O, Hogeveen H, van Weryn T, Nielen M (2009). Meta-analysis of dry cow management for dairy cattle. Part 1. Protection against new intramammary infections. J. Dairy Sci.

[11] Alekish M.O (2015). The association between the somatic cell count and isolated microorganisms during subclinical mastitis in heifers in Jordan. Vet. Med. (Praha).

[12] Al-Tarazi Y.H, Chakiso A.Y, Lafi S.Q (2011). Prevalence and distribution of bovine mastitis pathogens and their antimicrobial resistance in primiparous dairy heifers in Northern Jordan. Jordan J. Agric. Sci.

[13] Alekish M.O, Al-Qudah K, Al-Saleh A (2013). Prevalence of antimicrobial resistance among bacterial pathogens isolated from bovine mastitis in northern Jordan. Rev. Med. Vet.

[14] Ismail Z.B (2017). Molecular characteristics, antibiogram and prevalence of multi-drug resistant *Staphylococcus aureus* (MDRSA) isolated from milk obtained from culled dairy cows and from cows with acute clinical mastitis. Asian Pac. J. Trop. Biomed.

[15] Huijps K, Lam T.J.G, Hogeveen H (2008). Costs of mastitis: Facts and perception. J. Dairy Res.

[16] Unnerstad H.E, Lindberg A, Waller K.P, Ekman T, Artursson K, Nilsson-Ost M, Bengtsson B (2009). Microbial etiology of acute clinical mastitis and agent-specific risk factors. Vet. Microbiol.

[17] Persson Y, Nyman A.K, Gronlund-Andersson U (2011). Etiology and ¨antimicrobial susceptibility of udder pathogens from cases of subclinical mastitis in dairy cows in Sweden. Acta Vet. Scand.

[18] Awale M.M, Dudhatra G.B, Avinash K, Chauhan B.B, Kamani D.R, Modi C.M, Patel H.B, Mody S.K (2012). Bovine mastitis: A threat to economy. Open Access Sci. Rep.

[19] De Vliegher S, Fox L.K, Piepers S, McDougall S, Barkema H.W (2012). Invited review: Mastitis in dairy heifers: Nature of the disease, potential impact, prevention, and control. J. Dairy Sci.

[20] Borm A.A, Fox L.K, Leslie K.E, Hogan J.S, Andrew S.M, Moyes K.M, Oliver S.P, Schukken Y.H, Hancock D.D, Gaskins C.T, Owens W.E, Norman C (2006). Effects of prepartum intramammary antibiotic therapy on udder health, milk production, and reproductive performance in dairy heifers. J. Dairy Sci.

[21] Hagnestam C, Emanuelson U, Berglund B (2007). Yield losses associated with clinical mastitis occurring in different weeks of lactation. J. Dairy Sci.

[22] Sampimon O.C, De Vliegher S, Barkema H.W, Sol J, Lam T.J (2009). Effect of prepartum dry cow antibiotic treatment in dairy heifers on udder health and milk production. J. Dairy Sci.

